# Influence of Medical Representatives on Prescribing Practices in Mekelle, Northern Ethiopia

**DOI:** 10.1371/journal.pone.0156795

**Published:** 2016-06-15

**Authors:** Birhanu Demeke Workneh, Mehari Gebregergis Gebrehiwot, Tigist Assefa Bayo, Meles Tekie Gidey, Yared Belete Belay, Desalegn Mergiaw Tesfaye, Terefe Teshome Kassa

**Affiliations:** 1 Pharmacoepidemiology and Social Pharmacy Course and Research Unit, Department of Pharmacy, College of Health Sciences, Mekelle University, Mekelle, Ethiopia; 2 Department of Medical Laboratory, Ayder Referral Hospital, Mekelle, Ethiopia; Kenya Medical Research Institute - Wellcome Trust Research Programme, KENYA

## Abstract

**Background:**

Drug promotion by medical representatives is one of the factors that influence physicians’ prescribing decisions and choice of drugs.

**Objective:**

To assess the influence of medical representatives on prescribing practice of physicians in health facilities, Mekelle, Northern Ethiopia.

**Materials and Methods:**

Facility-based cross-sectional study was conducted enrolling all physicians working in public and private health facilities. All public and private health facilities were included and similarly, all physicians rendering services in these facilities were sampled in the study. The data were collected from February to March, 2015. Data were then entered into Epidata Version 3.1 and transferred to STATA version 12 for analysis. Both bivariable and multivariable logistic regressions were used to determine predictors.

**Results:**

Of the ninety physicians approached in this study, 40 (48.2%) of the physicians believed that their prescribing decisions were influenced by visits of medical representatives (MRs). The odds of physicians who received gifts from MRs being influenced to prescribe their respective products was six times higher than those who reported not accepting any gifts [AOR = 6.56, 95% CI: 2.25, 19.13]. Stationery materials 23(35.4%) and drug samples 20(54.2%) were the commonest kinds of gifts given to physicians and face to face talking 45(54.2%) was the most frequent promotional methods. The finding of this study showed that around thirty-nine percent of MRs have had negative attitude toward competitors’ product. Moreover, working in private health facility was also another predictor of influence of prescribing decision in the study area [AOR = 12.78, 95% CI: 1.31, 124.56].

**Conclusion:**

Nearly half of the physicians working in Mekelle reported that their prescribing decisions were influenced by MRs in the last 12 months. Accepting gifts and working in private health facilities were predictors of influencing prescribing decisions. However, most MRs fails to provide adequate and accurate information regarding their products and they had a negative attitude towards the competitors’ product(s).

## Background

World Health Organization (WHO) defines pharmaceutical promotion as “all information and persuasive activities by manufacturers and distributors, the effect of which is to induce the prescription, supply, purchase and/ or use of medicinal drugs” [1]. Drug promotion has a key role to stimulate prescription and sales of pharmaceuticals [2]. It has also an important impact on the rational use of pharmaceuticals, drug price-control mechanisms, the manufacture, availability and use of essential drugs, equity of drug distribution and the cost of health care system. It thus becomes a public health concern [3].

Pharmaceutical companies promote their product through their medical representative (MRs) by using drug sample, printed product literature and gifts that helps them to increase acceptability of their product [4]. Many prescribers receive pens, notepads and coffee mugs so that a targeted drug’s name stays uppermost in subconscious mind of the prescribers [5]. The interaction even extends from financial support of companies for research and medical education to the provision of up-to-date treatment details and educational materials [6].

It is known that different promotional strategies used by MRs in detailing their product and the quality of information provided in developing countries is poor compared to developed nations [7]. Even though there are WHO specifications in promotion of pharmaceuticals, most MRs fail to provide the necessary information for physician during their promotional activities. A study showed that 75.2% of physician agreed that MRs frequently used the word “safe” and only 19.7%, 20.4% and 23.6% of MRs explained regarding drug interaction, adverse drug reactions and cost of the drugs respectively [8].

Medical representatives visiting physicians is one of the factors that affect their prescribing practices [9]. The physician plays an important role in deciding which pharmaceutical brand is appropriate for patient’s condition so the main focus of pharmaceutical industry is to influence the decision making process of physicians [10]. Despite the fact that most physicians did not notice this influence, it is needless to say that MRs have substantial impact on their prescribing practice and selection of drugs. A study also revealed that physicians have a tendency for irrational prescribing, more favorable attitudes towards pharmaceutical companies, preference for newer, more expensive drugs and an inability to identify incorrect claims about medications [11].

Drug promotion is one of the condition that leads to prescription of expensive drugs in their brand names although National Drug Policy of Ethiopia encourages generic prescribing [12]. To the best of our knowledge, no study has described the effect of MRs on physician prescribing practices in Ethiopia, so this study will certainly provide baseline information about the extent of influence of MRs on prescribing decisions and practices of physicians. Hence, the aim of this study was to assess the influence of MRs on prescribing practice in Mekelle, Northern Ethiopia.

## Materials and Methods

### Study area and period

The study was conducted in both private and public health facilities in Mekelle. Mekelle is the capital city of Tigray region, at a distance of 783km towards North from the Ethiopian capital city, Addis Ababa. This study was conducted from February to March 2015.

### Study design and Sampling

A cross-sectional study design was employed to assess the influence of MRs on prescribing practice. All public and private health facilities in Mekelle were considered and all physicians working in public and private health facilities who volunteered to participate were included.

### Data Collection Techniques and Procedures

Data were collected using a structured, pre-tested, self-administered questionnaire. The questionnaires consisted of two parts. The first part contained questions about the socio-demographic characteristics of the physicians (age, gender. religion, average monthly income, year of experiences, qualification and practice sites). The second part contained questions about drug promotion and prescribing practice. The investigators administered the questionnaires to respondents and were also responsible in collecting the filled questionnaires. The questionnaire was developed in English language and on average it took about 10 minutes to complete.

### Data Quality Control, Management and Analysis

Codes were given to the completed questionnaires and then data were entered into Epidata Version 3.1 database and transferred to STATA version 12 statistical packages for analysis. Pretest of the questionnaire was carried out. Investigators had checked the filled questionnaires for their consistency, completeness and accuracy on daily basis. Cleaning of data was done to ensure the consistency and completeness of the data set. Both bivariable and multivariable logistic regressions were used to identify significant predictors. Frequencies, proportions and summary statistics were used to describe the study population in relation to independent variables whereas the logistic regression was employed to assess association between the different covariates and outcome variable. While building logistic regression model, we first conducted bivariable analysis using the various covariates and outcome variable (influence of MRs on prescribing practices). Consequently, those independent variables (practice sites, accepted gifts and attending sponsored-training) that showed association with outcome variable with p-value of less than or equal to0.3 were legible candidates to be considered in the multivariable logistic regressions. The degree of association between independent and dependent variables was assessed using odds ratio with 95% confidence interval.

### Ethical considerations

Ethical clearance was obtained from Research and Ethical Review Committee of College of Health Science, Mekelle University and then official letters of cooperation were provided to all private and public health facilities to get permission. Written consent was also secured from each study participant on voluntary basis prior to data collection. In the structured questionnaire, there were no questions that required personal information and the data from the returned questionnaire were assessed confidentially. The data were not made available to third party without permission from study participants.

## Results

### Socio demographic characteristics of the respondents

Out of 90 questionnaires distributed to respondents, 83 were filled completely with response rate of 92%. About three fourth (75.9%) of the respondents were males and 36 (43.4%) of them were within the age group of 31–39. Fifty two (62.7%) got 200–400 USD monthly and 63 (75.9%) of the respondents were within 1–5 years of service experience (Table 1).

**Table 1:** Socio-demographic characteristics of respondents (N = 83) in Mekelle, Northern Ethiopia, 2015.

Characteristics	Frequency(N)	Percent (95%CI)
Gender		
Males	63	75.9(65.6, 84.2)
Females	20	20.1(15.8, 34.4)
Age group		
22–30	34	41.0(30.3, 51.7)
31–39	36	43.4(32.8, 55.4)
40–48	10	12.0(5.0, 19.3)
= >49	3	3.6(0.0, 8.4)
Income category		
<200USD	14	16.9(7.5, 25.3)
200-400USD	52	62.7(50.0, 75.8)
>400USD	17	20.5(13.3, 27.7)
Experience		
< = 5	63	75.9(67.6, 84.2)
6–10	16	19.3(10.8, 26.5)
>10	4	4.8(1.2, 9.6)
Qualification		
GPs	52	62.7(53.2, 74.7)
Specialist	31	37.3(25.3, 46.8)
Religion		
Orthodox	57	68.7(56.6, 78.3)
Catholic	3	3.6(0.1, 8.4)
Protestant	9	10.8(4.8, 18.1)
Muslim	14	16.9(9.6, 25.2)
Practice site		
Public	50	60.2(49.4, 72.1)
Private	11	13.3(7.2, 23.8)
Both	22	26.2(18.1, 37.2)

### Drug Promotion and Prescribing Practices

Of all respondents, 81(97.6%) of them were visited by MRs for promotional purpose. A significant portion of physicians 32(38.6%) were visited occasionally while only 1(1.2%) physician was visited daily in the last 12 months. Almost half, 40(48.2%) physicians spent 2–10 minutes for discussion with drug promoters while only 10(12.0%) of physicians spent >21 minutes (Table 2).

**Table 2:** Frequency of visit and length discussion of drug promoter with physicians on the last 12 months (N = 83) in Mekelle, Northern Ethiopia, 2015.

Variables	Frequency(N)	Percent (95%CI)
**Frequency of visit**		
Daily	1	1.2 (0.0,3.6)
2-3/week	8	9.6 (1.3, 15.7)
1/week	15	18.1(8.4,24.1)
2/month	25	30.1(18.1,43.2)
Occasionally	32	38.6(25.6, 50.6)
Never	2	4.8 (1.2,9.5)
**Length of discussion (minutes)**		
Less than or equal to 10	40	48.2(36.3, 61.3)
11–20	33	39.8(27.9, 50.6)
More than or equal to 21	10	12.0(7.2, 19.1)

**Fig 1:**
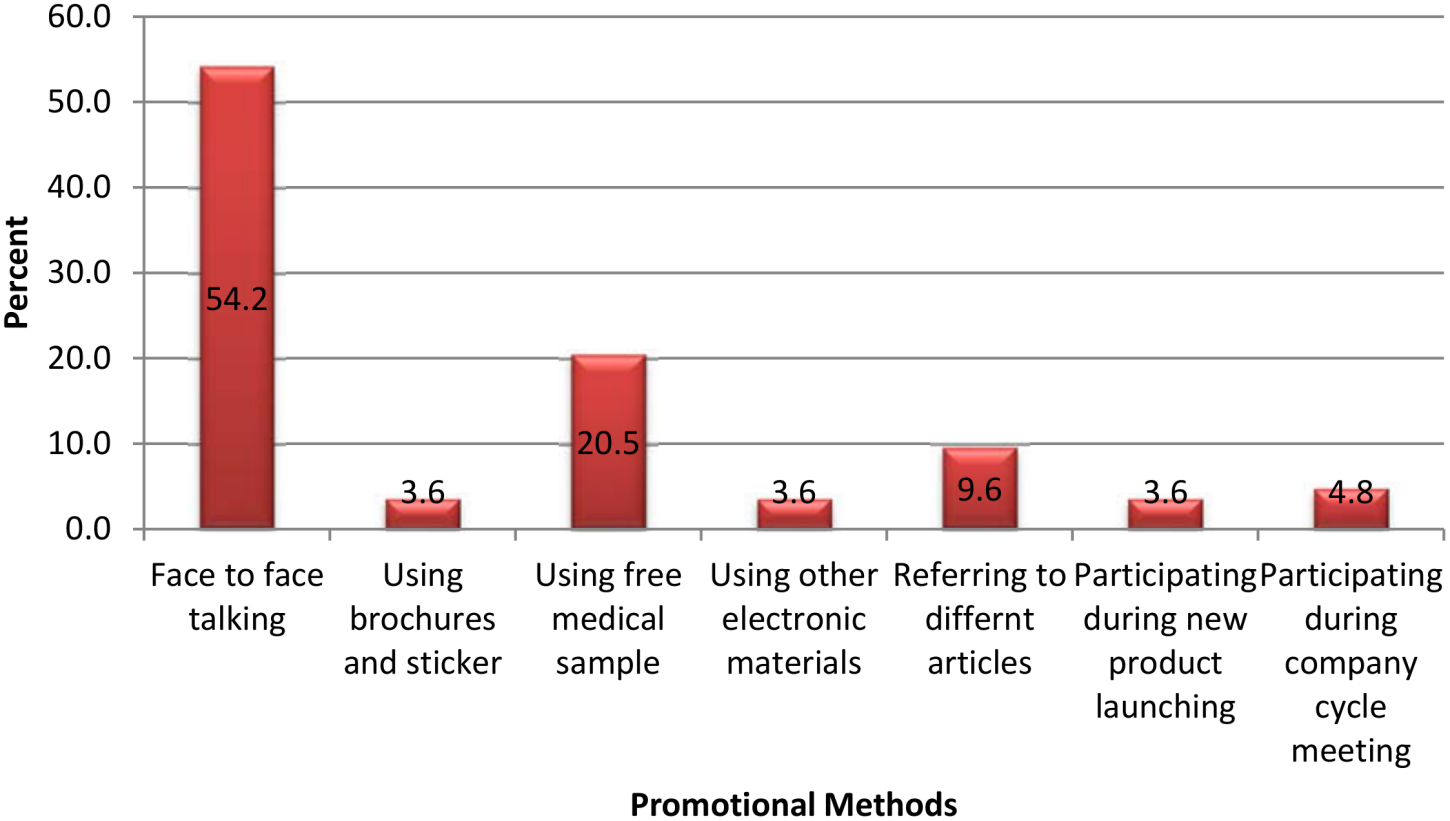
Promotional methods used by medical representatives in Mekelle, Northern Ethiopia, 2015.

More than half of drug promotions were carried out by face to face talk whereas referring electronic media was least commonly used promotional methods (Fig 1).

Almost half, 42(50.6%) of the respondents accepted gifts from drug promoters in the last 12 months while more than one fifth, of physicians were accepted gifts frequently. Office stationery, 23(35.4%) were more frequently accepted gifts. In this survey, 20(24.1%) of the respondents were participated in company sponsored-training program (Table 3).

**Table 3:** Frequency and kinds of gifts offered to physicians in Mekelle, Northern Ethiopia, 2015 (N = 83).

Characteristics	Frequency(N)	Percent(95% CI)
Accepted gifts		
Yes	42	50.6(34.0, 62.7)
No	41	49.4(37.3, 66.0)
Frequency of gifts		
Always	2	2.4(0.0, 6.0)
Frequently	18	21.7(13.3, 33.5)
Occasionally	15	18.1(11.0, 26.5)
Rarely	7	8.4(2.6, 16.6)
Never	41	49.4(41.0, 59.0)
Kind of gifts		
Dinner invitation	11	17(6.0, 20.5)
Drug samples	20	30.7(16.9, 32.4)
Stationery	23	35.4(18.1,37.2)
Coffee cups	8	12.3(3.8, 16.7)
Sponsored education	3	4.6(0.0, 8.4)

Our study revealed 40 (48.2%) of the physicians believed that their prescribing behaviors were influenced by visits of MRs although two third (65%) of the physicians were not satisfied in the current way of drug promotion. More than four fifth 70(84.3%) of information provided by medical representatives to physicians is about the brand name of a product followed by approved drug indication, 25(30.1%). On the contrary, the physician received scarce information on drug contraindications, interaction and precautions from MRs with 4.8%, 4.8% and 6% respectively. In addition, 53(63.9%) of physicians said that MRs had never informed them adequately and accurately (Table 4).

**Table 4:** Characteristics of drug information provided by medical representatives to physicians in Mekelle, Northern Ethiopia, 2015.

Variables	Frequency(N)	Percent(95%CI)
**Type of drug information**		
Approved drug indication	25	30.1(20.7, 39.4)
Brand name of the product	70	84.3(74.5, 93.8)
Drug interaction	4	4.8(1.2, 10.8)
Price	22	26.5(18.4, 37.8)
Side effects	6	7.2(2.4, 14.5)
Contraindications	4	4.8(1.2, 9.6)
Precautions	5	6.0(1.2, 12.1)
**Extents of adequacy & Accuracy of information**		
Always	4	4.8(0.0, 9.6)
Frequently	14	16.9(8.7, 27.4)
Occasionally	7	8.4(2.4,15.5)
Rarely	5	6.0(1.3, 12.0)
Never	53	63.9(53.2, 75.8)

Regarding focus promotional areas 43.4% physicians agreed that MRs gave their focus on selling their products. Only 3.6% of respondents believed that MRs focused on the scientific background of their product. This study revealed thirty two (38.6%) physicians believed that most drug promoters had negative attitude regarding competitor’s product (Table 5).

**Table 5:** Responses of physicians about area of focus and attitude of medical representatives regarding competitor’s product in Mekelle, Northern Ethiopia, 2015.

Variables	Frequency(N)	Percent(95% CI)
**Focus of MRs promotional area**		
Selling point of their product	36	43.4(32.5, 54.1)
Differential advantage of their product	32	38.6(30.1,48.1)
Scientific background of the product	3	3.6(0.0, 9.5)
Formulation advantage of their product	12	14.5(7.4, 20.5)
**Attitude of MRs towards competitors`products**		
They all had negative attitude	15	18.1(7.4, 25.3)
Most of them had negative attitude	32	38.6(27.7, 50.3)
Most of them were neutral	29	34.9(23.2, 45.6)
Almost none of them had negative attitude	7	8.4(3.6, 15.7)

The most frequent resources used in case of any problem in prescribing process were medical text books 47(56.6%) and academic journals 12(14.5%) (Table 6).

**Table 6:** References used by physicians in case of problem during prescribing process in Mekelle, Northern Ethiopia, 2015.

References used	Frequency(N)	Percent(95% CI)
Consultation of drug promoters	8	9.6(2.4, 15.7)
Drug guides of pharmaceutical company	6	7.2(1.2, 13.3)
Medical text books	47	56.6(47.0 67.5)
Academic journals	12	14.5(8.6, 21.4)
Consultation with specialist doctor	8	9.6(3.8, 15.7)
Consultation with other GPs	2	2.4(0.0, 6.0)

As shown in Table 7, acceptance of gifts remains most significant factor influencing physicians drug prescribing practice that is physicians accepting gifts from MRs were influenced six times more [AOR = 6.56, 95% CI: 2.25, 19.13 P = 0.001] compared to physicians who did not accept any gifts. Another factor influencing physician’s drug prescribing practice was working in private health facilities, [AOR = 12.78, 95% CI: 1.31, 124.56, p = 0.028] which means those working in the private health facilities were almost thirteen times more likely to be influenced into changing their prescription practices by MRs than those in public ones (Table 7).

**Table 7:** Predictors ofinfluence of MRs onprescribing practices of physicians in health facilities of Mekelle, Northern Ethiopia, 2015.

Characteristics	Influence on Prescribing Practices		COR (95% CI)	AOR (95% CI)	P Value
	No	Yes			
**Gifts**					
No	31	10	1	1	
Yes	12	30	7.75 [2.91–20.61]	6.56 [2.25–19.13]	0.001
**Practice sites**					
Public	32	18	1	1	
Private	1	10	17.78 [2.10–150.38]	12.78 [1.31–124.56]	0.028
Both	10	12	2.13 [0.77–5.91]	1.41 [0.44–4.55]	0.565
**Attending Sponsored-Training**					
No	37	26	1	1	
Yes	6	14	3.32 [1.13–9.78]	1.46 [0.40–5.27]	0.566

## Discussion

This cross-sectional study was done among 83 physicians working in private and public health facilities to assess the influence of drug promotion by MRs on physicians`prescribing decisions. Almost 98% of physicians were visited by MRs and of which nearly half (48.2%) of them reported that their prescribing decisions were influenced by the drug promotions. The provision of gifts and training sponsored by pharmaceutical company, mutual benefits between physicians and pharmaceutical company and working in private health facilities might be the probable reasons behind this relatively high figure.

The finding of this study was within a range of previously done similar studies. A study in Saudi Arabia reported 41.0% of the physicians`prescribing decisions were influenced by MRs [7]. Lack of on job training (OJT) on prescribing protocols and medical ethics in both private and public health facilities might be reasons behind higher degree of influence in prescribing decisions in our study. On the other hand, inclusion of only specialists and provision of OJT on prescribing practices in the Saudi study might be additional contributing factor for this difference. However, the result of the current study was significantly lower than studies done in Nigeria (60%) and Turkey (61.2%) [3, 13]. Less frequent visit by MRs and the doubtfulness of physician on the accuracy and adequacy of the information provided by MRs in this study could be the reasons for this variation. Our finding was also found to be inconsistent to the findings reported in German where the majority of doctors thought they were mostly immune to the drug industry’s attempts to influence them and accepts gifts [14].

One of the mechanisms used by pharmaceutical industry to promote their product and influence prescribing decisions is providing gifts to physicians. Whenever a physician accepts a gift, an implicit relationship is established between the doctor and the company resulting in a prescription [4]. Researchers described that there is, and will continue to be, an ongoing relationship of doctors with pharmaceutical industry that might affect decision making in the clinical context [15]. In our study, 50.6% of the respondents reported that they accepted gifts from MRs and of which, 21.7% of them were accepted gifts frequently. Office stationery (35.4%) were the most frequently accepted gifts followed by drug sample (30.7%) and dinner invitation (17%). A survey of German physicians similarly revealed that pharmaceutical samples, items of office stationery and free lunches were the most commonly received gifts [14]. Again, a study in Iraqi reported that offering gifts is a common cause for conflicts of interest for physicians that negatively influence prescribing patterns and result in early adoption to prescribe newly marketed medications depending on promotional information even in absence of clinical evidence about the drug effectiveness or side effects, which may result in undesirable outcomes to the patient [16]. In binary logistic regression analysis, physicians accepting gifts from MRs were six times more likely to get influenced than those who did not accept any gifts in their prescribing decisions. This gives an imperative message for MRs and pharmaceutical companies to choose the right promotional approach and tools to influence prescribing decisions of physicians.

Practice site can be another important factor affecting physicians for their prescribing decisions and practices and MRs visits and attempts to influence physicians’ prescribing behavior are a part of everyday life in private medical practice [14]. Researchers argued that mushrooming of drug companies is also responsible for this unethical medical practice. Since, one drug company in order to surpass another company brings out the same drug at much cheaper price, compromising the quality of the drug [17]. However, it is clear that national regulatory bodies and all other concerned parties should encourage pharmaceutical companies to exercise ethical marketing practices [18]. Physicians working in the private health facilities had the opportunity to frequently interact with MRs. The odds of physicians prescribing decision who worked in private health facilities alone were about 13 times influenced by MRs visit compared to those who worked in public ones. This difference might be due to the reason that public health institutions in Ethiopia are supplied their pharmaceutical consumptions by government agency, which were generic drugs. Accordingly, physicians prescribed available drugs in the stock regardless of what kinds of drugs were being promoted. On the other end, physicians working in the private health facilities would be more influenced as the practice in its nature entails business elements.

Most physicians referred medical text books as the main references than drug guide of pharmaceutical companies and consultation of MRs in case of any problem in prescribing process. This could be justified as MRs used face to face talking and product detailing as marketing techniques. Moreover, the common promotional tools in use were stationery materials and drug samples which did not contain relevant information to physicians beyond telling brand names of the products. In addition, 63.9% of the study participants claimed MRs had never informed them adequately and accurately about the drug being promoted. By contrast, 61% of those physicians who prescribed new drugs in German felt that they had often to always received adequate information and advice (14). It has been also reported that 52.5% physicians in Saudi Arabia used text books and Micromedex as reference resources while drug guides of MRs were the least frequent used resources (29.8%) [7].

In this study 84.3% of physicians reported that MRs provided information about the brand name of their product whereas about two- third of them responded that negative aspects of drug such as contraindications, precautions, drug interactions and major side effects of promoted products were never or partly answered during promotional visits. Nearly two third of the physicians were not satisfied with the current way of drug promotion even though only 28 (33.7%) agreed on its continuation. Previously done studies also relieved that MRs provided physicians with sufficient information about drug indication but rarely or never mentioned about negative aspects of the products [19,20,21]. In contrast, a study in Saudi reported 60% of physicians gained more information about a certain drug from MRs and 81.4% of them claimed that they confirmed the accuracy of the information given to them [7]. Differences in strength of regulatory control and supervision of MRs activity among countries might be the reason for this variability. This result indicates that the promotional undertakings weren’t in line with the guideline of Ethiopian Food, Medicine and Healthcare Administration and Control Authority. According to this guideline the information disseminated should at least contain name of active ingredients, brand name, content of active ingredient, indication, dosage form, side-effects, major adverse drug reactions, precautions, contra-indications, warnings, major drug interactions storage condition, and reference to scientific literature as appropriate. In addition, MRs are not allowed to detail the differential advantages of their products in reference to others [22].

As a weakness, the low sample size of this study (thought the study considers all the available study participants) would have its own negative implication about the power of the study to draw conclusions. We then recommend further study of similar protocol covering wider areas and greater number of study participants (larger sample size). In addition to this, the data collection was effected through self-administered questionnaires and all the data were self-reported and might be subjected to bias. The fact that this study adopted a cross sectional study design, investigating cause- effect relationships among outcome variable and its predictors was unlikely.

The key messages that authors would love to encode in this study are: one in every two physicians working in Mekelle reported that their prescribing decisions were influenced by MRs in the last 12 months. Accepting gifts and working in private health facilities were important predictors of these influences on physicians`prescribing practices. In addition, most MRs fails to provide adequate and accurate information regarding their products rather they focused on selling their products and most of them had negative attitude towards competitor products. The findings of this study, therefore, provided insights into possible target areas for drug policy makers and regulatory bodies in Ethiopia to shape the Drug Prescribing Policy and develop a comprehensive guideline for engagement of MRs with health care providers along with follow up mechanisms for its enforcement.
